# Garden Smellscape–Experiences of Plant Scents in a Nature-Based Intervention

**DOI:** 10.3389/fpsyg.2021.667957

**Published:** 2021-06-29

**Authors:** Anna María Pálsdóttir, Sara Spendrup, Lennart Mårtensson, Karin Wendin

**Affiliations:** ^1^Department of People and Society, Swedish University of Agricultural Sciences, Lomma, Sweden; ^2^Faculty of Natural Sciences, Kristianstad University, Kristianstad, Sweden; ^3^Department of Food Science, University of Copenhagen, Frederiksberg, Denmark

**Keywords:** pelargonium, horticulture therapy, odor, public health, ambient scent, stress-related mental disorder, well-being

## Abstract

This study explores how participants suffering from stress-related mental disorders describe their perception, interaction, and lived experience of garden smellscape during their nature-based rehabilitation. Natural elements, and especially nature smells, have been found to have a profound effect on stress reduction, suggesting an interesting link between odor in nature and stress reduction. The study was conducted as a longitudinal case-study, running over a period of 5 years, investigating participants’ perceptions of a garden smellscape, after completing a 12-weeks nature-based rehabilitation in Alnarp Rehabilitation Garden, Sweden. All participants were treated for stress-related mental disorders. Data were collected through retrospective semi-structured individual interviews and analyzed according to interpretative phenomenological analysis. The results revealed in what way nature odor (odor in nature) evoked associations, emotions, and physical reactions and provide examples of how nature scents function as a catalyst for sensory awareness and memories. Findings supported the understanding that experiencing the smell of plants, especially pelargonium, may facilitate stress reduction and support mental recovery in a real-life context. The results of the study can be used for several purposes; thus, they are relevant for actors within the development of nature-based therapy, as well as stakeholders within the horticultural industry.

## Introduction

Stress and stressful life situations can have a negative effect on a person’s executive functions (e.g., memory, cognitive performance, and mood) as well as sensory system, in terms of increased or reduced sensitivity (Jonsdottir et al., 2013). When exploring activities and treatments that are considered to alleviate the negative effects of stress, nature and natural elements have been identified as possessing qualities that may support well-being and mitigate the effects of stress. This link between nature and human well-being, especially the understanding of how different elements of nature underpin a positive impact, is of great interest. Several studies have explored the health benefits to people who reside in natural environments (Mitchell and Popham, 2008; Hartig et al., 2014; Frumkin et al., 2017); exploring how nature can contribute to restore cognitive functions and facilitate mental recovery (Ulrich et al., 1991; Van den Berg et al., 2010; Tyrväinen et al., 2014) as well as nature’s capacity to reduce stress (Ulrich et al., 1991; Sonntag-Öström et al., 2011; Corazon et al., 2019). Further, there are also promising evidences that nature exposure may have an influence in the decrease of inflammatory conditions when inhaling certain volatile natural compounds (Andersen et al., 2021).

Smellscape is a central concept when studying and exploring the holistic odor experience of a place, such as a garden. The term was described by Porteous(1990, pp. 21–45) as the totality of the olfactory landscape in a specific environment (Henshaw, 2014, p. 5). Recently, the concept of urban smellscape has been explored by Henshaw (2014) to understand the perception and experience in a real-life situation has explored the concept of urban smellscape. Henshaw covers different perspectives of olfactory stimuli but interestingly touches on the aspects of how to design restorative smellscapes. In a recent Virtual Reality (VR) study on urban smellscape, Hedblom et al. (2019) identified that odor has a greater impact on stress reduction than visual and auditory stimuli. They highlighted that smellscapes in urban settings are an important aspect for human health and well-being. Yet, the understanding of the mechanisms behind the identified phenomena, especially the significance of odor perception, is less well researched (Henshaw, 2014, p. 5; Hedblom et al., 2019).

In human evolution, the key to survival has been the sense of smell, as it can detect and signal danger or safety and trigger an instinctive and subconscious reaction to fight, flight, or stay (Porteous, 1943). The odor perception comprises (1) odor characteristics, (2) environmental, and (3) individual factors (Henshaw, 2014, p. 26). The odor characteristics depend on the rate of evaporation, the concentration, and intensity, as well as the trigeminal stimulation. The environmental factors such as air temperature and quality, the surface of the environment, and wind have a significant impact on the detection of the odor. The individual factors for identifying and detecting odor can vary depending on age, gender, and a person’s health status. Odor memory is formed from the very early days of our life and develops during our entire lifetime (Sugiyama et al., 2015). It has been shown that odors have a significant impact on memory, for example, the presence of odors can improve recognition (Dinh et al., 1999; Parker et al., 2001). Pointer and Bond (1998) suggest that context-dependent memory processes may underlie the formation and retrieval of odor-evoked memories, while Olofsson et al. (2016) have shown links between odor and memory due to genetics, pointing out the ϵ4 allele of the Apolipoprotein E gene as a risk factor. Köster et al. (2014) point out that unexpected experience of odors are easy to note and to remember.

Further, Olofsson et al. (2020) showed that the olfactory system was highly responsive to training and could promote cross-sensory transfer by increasing the visual learning. It has also been shown that exposure to odors, both outdoor and in-door odors, may enhance the olfactory function as well as odor memory (Mahmut et al., 2020b). There are also reports showing that loss of olfactory sense is associated with cognitive decline and dementia (Behrman et al., 2014; Stanciu et al., 2014). The decline of the *olfactory sense*, i.e., perception of odors and flavors, has been connected to age, different diseases, and mental health in previous studies (Murphy, 2002; Hummel et al., 2007; Bahar-Fuchs et al., 2011). However, Mahmut et al. (2020a) suggest that mindfulness training may improve perception of odors. The individual differences have been proven to be large, and the decline is often larger in men than in women (Hummel et al., 2002). However, studies suggest that the heterogeneity in olfactory decline is often related to secondary factors such as medication and dental health (Nordin et al., 2012; Sulmont-Rossé et al., 2015). Recent findings suggest that the age-related decline in olfactory sensitivity is not uniform but rather odor specific, as identification of mushroom-like odor and cinnamon was found to be equally identified across all ages (Seow et al., 2016).

The olfactory receptors are found in a small part of the epithelium, which is in our nasal cavity. These receptors are activated by volatile chemical molecules that stimulate the olfactory receptors, either *orthonasally*–through the nose when we smell–or *retronasally*, i.e., via the mouth, where the “odor molecules” are released from the food or beverage when we chew and thus contribute to multisensory flavor perception when we eat (Albinsson et al., 2017; Wolfe et al., 2017). Natural odors, such as, e.g., *Convallaria majalis* L. (Liliaceae), *Jasminum sambac* L. (Oleaceae), *Rosa x alba* L. (Rosaceae), and Chrysanthemum, that have been perceived as pleasant can evoke the feeling of joy, improve mood (Weber and Heuberger, 2008; Francisca et al., 2019) and have a calming effect on one’s mind (Pálsdóttir et al., 2014; Sidenius et al., 2017). Olfactory properties of essential oils such as lavender, rosemary, and chamomile have been suggested to have a positive effect on mood and objective cognitive performance (Toda and Morimoto, 2008; Moss et al., 2009), which is in line with findings suggesting the stress-reducing effects, in what is more holistically described as, e.g., “green odors” (Fujita et al., 2010). Natural odors are also a central part of the multi-sensory experience and nature-based intervention called forest bathing in conifer forest, which presently is being used in Japan, to improve human health and reduce stress (Tsunetsugu et al., 2010). The positive effects of a coniferous forest are also identified in other studies where a walk or stay in the coniferous forest showed increased well-being and reduce stress level (Park et al., 2010; Dolling et al., 2017; Li, 2019). When separating the visual, auditory, and olfactory nature stimuli on stress reduction (such as feeling calm and relaxed), smells seem to have a more profound effect on stress reduction than visual and auditory stimuli (Hedblom et al., 2019), suggesting an interesting link between smells of nature and stress reduction. Due to lack of research in life world context the aim of the study was to investigate how participants in nature-based rehabilitation describe their perceptions and lived experiences of a garden smellscape.

## Materials and Methods

The study was conducted as a longitudinal single case-study (Yin, 2009), running over a period of 5 years, investigating participants’ perceptions of a garden smellscape, after completing a 12-weeks nature-based intervention in Alnarp Rehabilitation Garden, Sweden. It should be notified that this study differs from aromatherapy by not including aromatic oils, topical applications, massage or inhalations but perceived plant odor in a garden context.

### Participants

All participants were treated for stress-related mental disorders, i.e., exhaustion disorder (ICD- F43.8a), or depression (ICD-F32.0 and ICD-F32.1). The exclusion criterion to take part in the nature-based intervention was known alcohol or drug abuse. The participants were Swedish residents, mean age 45.5 years (25–62 years), with various levels of educational and professional status. Altogether, 59 former participants (50 women: nine men) participated in the study.

### Procedure

#### Nature-Based Intervention

Since 2002, the Swedish University of Agricultural Sciences has conducted innovation projects on nature-based intervention at SLU Alnarp Rehabilitation Garden (Stigsdotter, 2003; Adevi, 2012; Tenngart Ivarsson and Grahn, 2012). In this context, nature-based intervention is defined as a health intervention (World Health Organization [WHO], 2021), implemented in an outdoor setting dominated by natural elements (Grahn et al., 2010; Corazon, 2012; Pálsdóttir et al., 2018a; Kristjánsdóttir et al., 2020; Segal et al., 2021) to support the rehabilitation process of individuals suffering from stress-related mental disorders, mainly exhaustion disorders (ICD 10 F42.8) (Gliese, 2014, pp. 13–15). The intervention was performed as a 12-weeks rehabilitation program supporting the participant’s rehabilitation process.

Each intervention group consisted of up to eight individuals (Pálsdóttir, 2016) including four treatment groups each year, i.e., (1) fall-winter, (2) winter-spring, (3) spring-summer, and (4) summer-fall. In order to cover each season at least three times, the study was run over a period of 5 years. An interdisciplinary team of four professions ran the rehabilitation program: an occupational therapist, a physiotherapist, a medical doctor specialist in mental health, and a horticulturist. The program ran 4 days a week for four hours each day and included rehabilitation sessions such as horticulture therapy, psychotherapy, occupational therapy, and physiotherapy (Pálsdóttir et al., 2013). The rehabilitation programs offered activities for both work and rest, all of which intended to stimulate the participants’ sensory experience. Each day was divided into four main sections–all activities were performed outdoors or in the greenhouses and in extreme weathers indoors: (1) morning gathering with herbal tea brewed on different kind of herbs (different kind of odors); (2) relaxation; (3) garden/horticultural activities and therapeutic sessions; and finally, (4) gathering and closure for the day (Pálsdóttir, 2014, pp. 35–36; Pálsdóttir, 2016).

**FIGURE 1:**
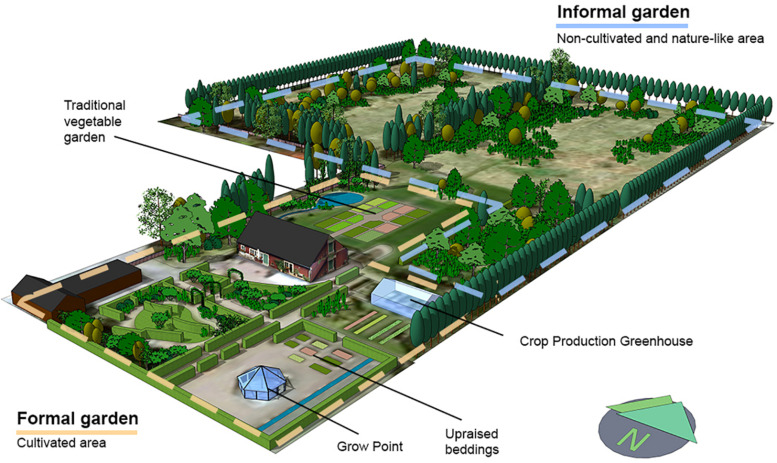
Alnarp Rehabilitation Garden (2 ha) is divided into two main areas; the formal and cultivated garden (marked with yellow a dotted line) and the nature area (marked with a blue dotted line). Illustration by Cerwén G. 2021.

The 2 ha garden was divided into two main areas: (1) the cultivation and garden area, a formal and cultivated space. The area had more of a garden or park-like character and (2) the nature area, a non-cultivated area with an informal appearance, including free growing vegetation and trees. The area had more of a natural characteristic (Figure 1). The vegetation in the garden included broad-leaf and evergreen trees, shrubs, perennials, and annuals as well as spring and fall bulbs, all for stimulating the different senses during the different seasons. The plant selection for the garden was based on using familiar plants (in Swedish context) that were easy to propagate and inexpensive. Attention was paid to visual aesthetics, i.e., different shapes, forms, and colors as well as olfactory and texture aesthetics, i.e., scented and tactile plants. There were places in the garden where one could engage in horticultural activities together with other participants or on their own, as well as places where one could sit and enjoy the surroundings (Pálsdóttir et al., 2018b).

**FIGURE 2:**
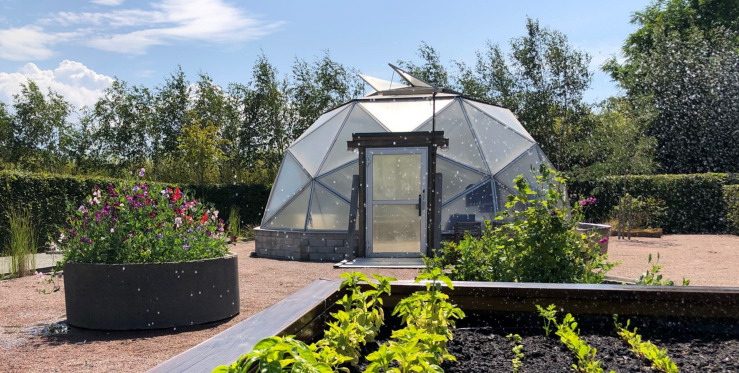
“Grow Point,” a domed greenhouse of 49 m^2^ used for storage of vegetative plants (e.g., pelargonium) and for early-stage propagation of seedlings and cuttings.

The formal garden area included a large traditional vegetable garden of 300 m^2^ as well as a smaller area with raised beddings to lift the crops closer toward the participants’ face to enjoy the odor and the taste of the crops. Also, there were two greenhouses, one production house of 100 m^2^ and one domed greenhouse “grow point” of 49 m^2^ for potted vegetated plants and for early-stage propagation of seedlings and cuttings (Figure 2).

The “grow point” greenhouse was kept heated during winter for storing semi-hard plants. At the time of the study, the pelargonium collection was vast and included both scented as well as flowering varieties. Pelargonium sp. is an established cultivated plant, and the most produced potted plant in Sweden. Pelargonium sp. has a characteristic scent of geraniol, citronellol, mentol, and eucalyptol, compounds found both in the tentacles on the stems and leaves (Martinsson, 2000, p. 158). During winter, the greenhouse was full of rosemary (*Rosmarinus Officianlis L*.), lavender (*Lavendula* sp.), verbena (*Aloysia* sp.), and ground cherries (*Physalis* sp.) potted plants in addition to the geranium (*Pelargonium* sp.) for winter storage. The greenhouse was open to the participants, who were welcome to rest or work with the plants as they wished. The participants could engage in tasks on their own or together with others, e.g., taking cuttings, watering the plants, trimming and removing dead leaves and over bloomed flowers. At the beginning of the intervention, the head gardener showed the participants how to take care of the plants, so they would be comfortable doing this on their own.

Each morning, the daily program started with a gathering and drinking herbal tea. The tea was either brewed with fresh or dried plant leaves harvested from the garden. Often, the tea was made with fresh leaves from one of the geranium plants, as it was possible to harvest during all seasons. The head gardener showed the participants the different plants as well as how to propagate them by either cutting or by seeds. The tea was often made using leaves from pelargonium (e.g., different varieties *Pelargonium* spp.; *Pelargonium × domesticum*; *Pelargonium × hortum; Pelargonium graviolens*, *P*. *graviolens “Dr*. *Westerlund;” Pelargonium crispum*, and *Pelargonium odoratissiumum*) and of menthe (*Mentha × piperita* L. and *Mentha* spp.). Sometimes other plants were also used to make the tea, such as rosemary, lemon verbena (*Aloysia Citrodora*), marigold (*Tagetes* spp.), and pot marigold (*Calendula Officinalis L*.). Besides using the leaves for the tea, the plants were also harvested for other types of consumption, e.g., cakes, pesto, and herb salts.

### Data Collection and Ethics Statement

All data are handled in accordance with the recommendations of the Swedish Ethical Review Authority for similar studies, and the study also follows SLU’s data handling policy, in accordance with the General Data Protection Regulation. Data were collected from December 2007 until fall 2012. After each intervention group (four each year), the participants were asked to give their informed consent if they wanted to participate in an individual interview. All participants could withdraw from the study at any given point without further explanation as well as pass on questions if they did not want to reply to the questions. The participation was anonymous. The study was conducted as a retrospective semi-structured individual interview study. The focus of the interview was the lived experience of the nature-based intervention and on the role of the nature/garden for the participants’ recovery process (Pálsdóttir, 2014; Pálsdóttir et al., 2014, 2018b; Cerwén et al., 2016). The first author conducted the interviews, within a month after the participants had ended their 12-weeks rehabilitation; all interviews took place in the rehabilitation garden in Alnarp. Each interview lasted for about 1 hour and was recorded, following the informants’ signed consent, and afterward transcribed for the analysis.

### Data Analysis

The 59 transcribed interviews were systematically searched to extract all aspects of odor mentioned in the interviews. As the interviews were conducted in Swedish, the search was conducted with Swedish terms related to scent, smell, olfactory, and odors and specific scented plants and herbs.

When each of the word was detected, the relevant surrounding text was read to collect the whole section where the topic was mentioned. All the sections mentioning the participants’ experiences relating to odor were collected into one document and analyzed according to stepwise procedure of interpretative phenomenological analysis (Smith et al., 1999, 2009). Interpretative phenomenological analysis aims to explore the participants’ lived experience of a certain phenomenon, i.e., the perceived and lived experience of a garden smellscape in the context of nature-based rehabilitation.

The first and the second author, independently read the text several times to get a sense of the whole content, making notes on the aspects of interest related to the study aim. Then the main themes were abstracted and clustered so superordinate themes emerged. At the end of this process, the two authors compared, discussed, and arrived at an agreement on the main themes. Finally, all four authors discussed and agreed on the final formulation of the main themes grounded in the participants’ narratives (raw data). The results are presented anonymously at a group level.

## Results

Four superordinate themes emerged, describing the participants’ perceptions and lived experience of the Alnarp Rehabilitation Garden smellscape. The results related to both the indoor (green house) and outdoor nature (the garden) experience and environments, see Figure 1. The plants referred to by the participants thus cover both traditional potted plants, vegetables, annuals, perennials, herbs, semi-woody shrubs, and fruit trees as well as nature in general. Besides the four main themes, one specific odor was mentioned more often than others, i.e., the odor from *Pelargonium* sp. Therefore, a special attention was given to the use of this species.

### Nature Scent–Associations, Emotions, and Physical Reactions

In general, the participants’ associations with nature scents were positive and expressed in terms such as nice smell, appreciation for natural fragrances, contributing to stress reduction and the feeling of being calm and relaxed, happiness, and joy of life. “*I go and feel the plants but also just stop and stand and just take in the plants*, *take in the place*, *the peace that is there and yes*, *breathe it in*, *you can say*.” Participants also expressed how nature scents increased the perceived closeness to nature, sensuality, and what was described as an appreciated disconnection of the intellect and connection to the body and mind. The participants mentioned many kinds of plants and plant material that they liked the smell of, such as the odor of herbs, fruits, flowers, leaves, and needles to dried straw and wood. All those odors were associated with pleasantness, feeling calm and happy, as well as the feeling of being present in the moment. All of which can contribute to stress reduction. The connectedness to nature through olfactory stimuli was expressed as reconnecting with the inner self, feeling alive and happy. One species, in particular, was mentioned more than others and with more descriptions of pleasant feelings and feeling calm when touching the leaves and smelling the odor, i.e., *Pelargonium* sp. was often mentioned in connection with the smell of citrus. Some varieties did not smell pleasant but were still appreciated as a sensory stimulus and fun to compare with the other pleasant-smelling plants.

Apart from expressing associations relating to specific plants (e.g., pelargonium), the concept of more holistic sensory descriptions such as the odor of “green house” and “garden” odor emerged several times. The participants had positive associations to soil and planting, both in indoor and outdoor plant related activities. Additional examples of participants expressing associations with nature scents include spring, being outdoors, “breathe in the plants,” and safety. Participants also described physical reactions in relation to lemon-scented plants, such as *“a kick for the brain*” (e.g., P*elargonium*, *Thymus*, and *Aloysia*).

It also became clear that the conversations about smells gave rise to reflections on natural and unnatural odors. The odors represented in the greenhouse, garden, or nature were described as natural scents, which participants separated from artificial smells. Unlike natural smells, artificial odors were described as unpleasant and sharp. Perfume was compared to the smell of cigarettes, viewed as pollution, intrusive, and leading to people taking up too much space. They evoked negative emotions, such as stressful and irritating. Despite the overall positive associations to nature scents, some participants also raised negative aspects connected to nature smells, such as being allergic or just simply not appreciating the smell of, e.g., pelargonium, due to what was described as its compact and too rich smell.

### Natural/Nature Smell Functioning as a Catalyst for Sensory Awareness and Memories

Several participants expressed how they interact with plants and touch them, often with the aim to feel the smell of the plant and achieve the feeling of calm.

*“*…*felt when I sat there and it was to touch all these spices*, *it will be the smells*, *it is the senses*. *Just that you get started a little*, *you are triggered to get started*.*”*

Participants who had experienced hypersensitivity and had difficulties in using their senses and experiencing smells, sounds, or taste expressed that nature scents could function as a catalyst to sensory awareness. *“Because I have not had my senses activated before*. *I did not see anything*, *I did not smell anything…”* Regardless of whether the person felt a lack of sensory experiences, or that the sensory experiences were too strong, natural scents seemed to be perceived as detectable or appropriate and tolerable. The sensory experience of plants was also related to existential issues such as being alive: *“I was so aware of how the grass smelled and I had also started to feel like a little more alive there*.*”* Participants who felt stressed expressed a desire to approach the plants, i.e., to smell and touch them, activities and sensory experiences that were experienced as relieving the stress. This was frequently and intentionally used as a medium for stress reduction, first practiced when in the rehabilitation program in the garden and then later brought into their home environment.

The participants produced their own plants by cuttings and selected their favorites to bring back home, both as a memory of Alnarp and to have their own sensory garden to use when they felt stressed and needed calming, especially some of the lemon/citrus scented geranium varieties. Several participants described how natural smells such as green house, soil, and straw evoked positive memories from childhood to life. The olfactory sense functions as a link, bringing happiness from childhood to the present.

“*It reminds me of when I was with my grandmother in her garden when I was a child*, *went there in the garden paths and her little greenhouse they had behind their little cottage and so*, *it was a very happy period as well”*.

Often, the positive memories related to time with grandparents in the garden or in the forest and helped the participants to reconnect with the feeling of joyfulness. The odor memories also helped the participants to recollect pleasant scents they used when needing their moods to be uplifted. The participants also expressed how unnatural smells brought back negative memories, such as stressful situations at work (perfume) and being a patient in a hospital (the smell of hospitals). Also, scents that signaled danger such as smoke and fire evoked anxiety in the participants. Further, they described how sensory stimuli was possible in nature but not in the indoor environment at the healthcare units. There was nothing there that could positively stimulate their senses compared to what was possible in the garden.

### The Seasonal Variation of Smell

Participants expressed that there was a special smell in the green house which appeared to be intense and at times almost overwhelming, especially in combination with high temperatures. This was not the case for smells from the outdoor environment. It was also obvious that the sensory experiences varied during the different seasons, and depending on the time of the year, the natural smells were different. One patient expressed a sense of melancholy when approaching apples during late fall. The patient viewed the natural elements (an apple) and its state of degradation as a parable of the self.

*“All the different varieties that smell and taste different… maybe there was some kind of melancholy in that total decay and which rhymed well with my mood*, *maybe because I was also mismanaged and decayed*.*”*

Participants who were undergoing treatment during the winter also expressed not being able to have a sensory experience of the outdoor plants during winter. During winter, the smells are primarily related to the scent of plants in the green houses, especially the geranium in the grow point greenhouse.

### The Fascination With Pelargonium

Since the most frequently mentioned plant species, concerning smell, was *Pelargonium* sp., especially the odor connected to citrus, we paid special attention to this species. Participants expressed a special interest in scented pelargonium varieties, in which both the leaves and the flowers gave odors, described as apple, peppermint, orange, rose, eucalyptus, and citrus. The participants liked to experiment with the different odors, rubbing the leaves and then smelling the best and the least pleasant scent. This experiment was mostly done together with others but when enjoying the favorite smell, it was done in solitude. During the program, the participants learned about different plants, how to propagate and cultivate them, and how to use/consume them. This interaction awakened an interest in keeping and cultivating plants in the garden as well as at home.

Several participants mentioned their pelargonium collection at home as their “Alnarp plants,” which made it possible for them to feel the same calmness at home as felt in the rehabilitation garden at Alnarp. The olfactory and visual pleasantness were the main reasons for keeping their pelargonium collection at home. Other reasons were the tactile experience of holding the leaves in their hands and brewing tea. At the time of the study, one species of pelargonium, Dr. Westerlund, was found in abundance at the location.

## Discussion

**FIGURE 3:**
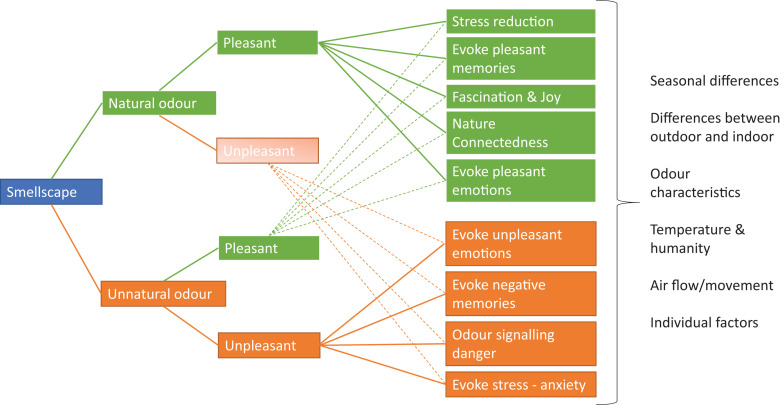
Illustration of olfactory mechanism identified as natural and unnatural odor.

The exploration of the perceived smellscape in the nature-based rehabilitation garden revealed four main themes concerning nature scents: (1) associations, emotions, and physical reactions; (2) Natural/nature scent functioning as a catalyst for sensory awareness and memories; (3) seasonal variation of scents; and finally, (4) the fascination with pelargonium. Identified themes provide example of several olfactory mechanisms that can be found in the garden context (natural scent) and outside of the garden context (unnatural scent) (Figure 3). The participants distinguished between natural odors and artificial odors, with the latter being associated with negative emotions, memories, and often stressful events. The natural odors were dominantly reported to evoke positive emotions, memories, and being joyful as well as stress reducing.

Several experimental studies have identified nature scents as important sensory stimuli, supporting stress reduction (Toda and Morimoto, 2008; Fujita et al., 2010), as well as there are odors inducing feelings of safety and comfort (Köster et al., 2014). Only a few studies have been conducted in a real-life context of lived experiences, particularly in a rehabilitation context.

It has been shown that odors affect eating behavior and seem to determine appetite and food choices, and thereby health (Hoover et al., 2020). In a recent study by Hedblom et al. (2019), implemented in a multisensory VR environment, the researchers found that a high rating of forest smell (olfactory stimuli: fir and mushroom) and park environment (olfactory stimuli: grass) resulted in a lower psychological stress response compared to an urban environment with olfactory stimuli of gunpowder and diesel. The perceived pleasantness of visual, olfactory, and auditory experiences at three different virtual environments (park, forest, and city) significantly differed, pointing out the olfactory sensory (scent) experience as the most important stimuli for stress reduction. The current study provides examples of how the act of handling and experiencing the smell of plants may facilitate stress reduction and support mental recovery in a real-life context (the Alnarp Rehabilitation Garden). Participants also expressed that natural olfactory stimuli (soil, wood, dried straw, vegetation) led to mental as well as physical relaxation. These were experiences that evoked feelings of being calm and happy, which is in line with effortless fascination as a source of mental restoration, as described by (Kaplan et al., 1989).

In the present study, natural smells such as lavender, grass, wood, and conifer needles were mentioned as pleasant and evoking strong feelings of connectedness to nature. Also, the feeling of being outdoors and breathing in the plants were associated with being part of nature. Nature connectedness is an eudemonic aspect of human well-being and has been positively correlated with a feeling of mindfulness and feeling happy (Howell et al., 2013; Capaldi et al., 2014). The participants described this as reconnecting with their inner self, feeling alive and happy. These feelings of being happy brought back positive memories from childhood (Proustian memories) and reconnected to feelings of joyfulness. The pleasant olfactory stimuli seemed to have a positive impact on the participants’ mood and also brought back happy memories and emotions from the past. Individuals suffering from stress-related disorders can have a dysfunctional sensory system, in terms of increased or reduced sensitivity (Jonsdottir et al., 2013). In such cases, they either sense little or nothing from the smellscape around them or are hypersensitive. Regardless of these conditions, the participants in the study seemingly perceived the natural smells as being tolerable, whereas the unnatural smells were less tolerable. It turned out that unnatural/artificial scents were often referred to as unpleasant and evoked both negative memories and emotions from the workplace and hospital environment, as illustrated in Figure 3. Olfactory stimuli such as from perfume, smell of cigarettes, and “hospital scent” were associated with stressful situations, being harsh and unpleasant, and associated with being a patient. In comparison with the indoor environment (workplace and hospital), the garden milieu was perceived as a place offering positive sensory stimuli, supporting their well-being. Pelargonium/geranium were mentioned repeatedly and perceived as popular among the participants. The results specifically indicate a specific positive link between sensory odor experiences with pelargoniums, described as rose, lemon, and citrus, and stress reduction as the participants intentionally used the smell of plants for stress reduction, both in the garden and then at home. Even though the location provided a vast variation of pelargonium scents, one was mentioned numerous times, i.e., pelargonium “Graveolens,” particularly a variety called “Dr. Westerlunds.” The explanation for this is partly that it had been identified as possessing a well-liked citrus odor and that due to plant physiological properties, it is easy to propagate which makes it suitable for implementing in activities within the program. Taking this into consideration, it is reasonable to assume that a great proportion of identified associations and expressed experiences with geranium, in general, are in fact closely linked to Dr. Westerlund. Previous studies exploring geranium have pointed out its health-promoting effects (Peace Rhind, 2014, pp. 304–305) and already during the 19th century, Dr. Ernst Westerlund (Swedish physician) recommended pelargonium (*P*. *graviolens)* for cleaning the air from diseases (Martinsson, 2000, pp. 164–167). More recently, the smell pelargonium has been identified as reducing anxiety (Morris et al., 1995) as well as enhancing relaxation and improving sleep (Spence, 2020). These findings support the results of the study; however, future studies are needed to get a better understanding and clarity on the subject.

### Future Studies

It was interesting to note the great fascination with pelargonium/geranium, and its stress reducing effects. Considering the great presence of the variety Dr. Westerlund, and that this variety has been studied scientifically in the past, it is reasonable to use this variety as a starting point for future experiments. However, in order to fully understand the mechanisms underpinning this effect, future studies should focus on identifying chemical compounds of pelargonium and the sensory perception of these compounds. Such knowledge is of great importance in knowing what nature odors may induce the perceived feeling of being relaxed and calm. A compilation of the geranium’s fragrance properties can be of great interest and benefit to the public, researchers as well as stakeholders and growers within the horticultural industry. Results such as those presented in this study, as well as future in-depth analysis and scientific grounding of the chemical aspects and other specific properties of plants, can further contribute to an increased interest in plant scents and how they affect humans. An effective plant aroma is for example lavender; it has been used in medicine as a narcotic, anti-inflammatory, and antidepressant substance (Khalil et al., 2019). It has for example been shown that lavender aroma was effective in reducing anxiety among cancer patients (Abbaszadeh et al., 2020). In lavender, the substances linalool and linalool acetate may stimulate the parasympathetic system, causing narcotic effects (Karadag et al., 2017).

## Conclusion

The study provides answers to what role scented plants play in nature-based rehabilitation for participants suffering from stress-related mental disorder. Finally, the result adds knowledge regarding whether there is a particular species, citrus scented pelargonium, which may reduce stress and support mental recovery more than others, specifically, its qualities. The results of the study can be used for several purposes, depending on the target group, and thus become relevant for actors within the development of nature-based therapies, but also firms and stakeholders within the horticultural industry, e.g., growers, nurseries, and producers of raw materials to produce plant extracts and geranium products. This indicates a potential for further development of novel products for stress reduction by using nature-based compounds or the plant itself.

## Limitations

Although the study was conducted over 5 years, including 59 representatives from all intervention groups during that time, the study only focused on one target group, i.e., individuals suffering from stress-related disorders. We acknowledge that other parts of the nature-based intervention program also contributed to stress reduction but in this study, we wanted to highlight the role of scent garden context. Therefore, we do not acclaim that overall stress reduction is solemnly due to scent experience. It is additionally important to highlight that the interviews are based on the participant’s narratives of how, e.g., plants have affected their mood and/or emotions. Since the participants not specifically were requested to consciously discuss alternative reasons to these perceived emotions/mood states we can’t claim with complete accuracy that the perceived effects always were caused by, e.g., plants. In order to increase the ecological validity of studies on lived experience and perception of a garden smellscape, other target groups should be included in future studies, both individuals participating in health interventions and the public. We found the use of interpretative phenomenological analysis an appropriate choice of method as it aims to explore in detail individual experience of a specific phenomenon, in this case, their experience of scent in the context of nature-based rehabilitation. However, the method is criticized for lacking standardization and fundamentally being a subjective approach suggesting other researchers might come up with a different interpretation of participants’ narratives.

## Data Availability Statement

The raw data supporting the conclusions of this article will be made available by the authors, without undue reservation.

## Ethics Statement

The studies involving human participants were reviewed by the Region Ethical Committee in Lund, Sweden. The patients/participants provided their written informed consent to participate in this study.

## Author Contributions

AMP contributed with the acquisition of data, analysis and interpretation of data, and drafted the initial manuscript and Figure 3. SS contributed with the analysis and interpretation of the data, and revised the article critically for important intellectual content. LM and KW revised the article critically for important intellectual content. All authors contributed to the conception and design of the study, including revision, reading, and approving the submitted version.

## Conflict of Interest

The authors declare that the research was conducted in the absence of any commercial or financial relationships that could be construed as a potential conflict of interest. The reviewer PM declared a shared affiliation, with no collaboration, with one of the authors KW to the handling editor at the time of the review.
